# T2 relaxation time mapping in phantom and in vivo myocardial studies to investigate optimal method of quantification

**DOI:** 10.1186/1532-429X-14-S1-P287

**Published:** 2012-02-01

**Authors:** Ina Vernikouskaya, Peter Bernhardt, Wolfgang Rottbauer, Volker Rasche

**Affiliations:** 1 Internal Medicine, University Hospital of Ulm, Ulm, Germany

## Summary

Reproducible and accurate T2 measurements are required for the characterization of myocardial tissue, e.g. to distinguish healthy, scar and edematous myocardium. It is the objective of this study to investigate the impact of the fitting algorithm applied for the T2 quantification from data derived from a single-breathhold T2-prepared steady-state free precession (T2p-SSFP) imaging technique.

## Background

In direct comparison to multi spin echo technique, superior performance of T2p-SSFP-based T2 quantification has been reported. In spite of availability of a variety of quantification algorithms, the use of mono-exponential model is still the standard for fitting the T2 decay curve.

## Methods

**Figure 1:**
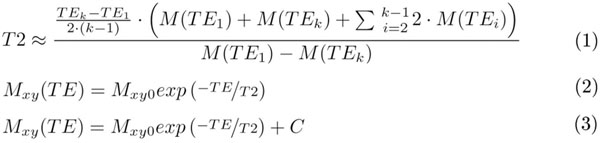
Models for fitting the T2 decay curve: (1) numerical approach, where *M* is the MR signal at a given echo time *TE*, (2) mono-exponential model, where *M_xy0_* is the total magnetization at *TE* = 0, (3) offset model with *C* modeling noise and artifacts.

3 different fit-models were investigated: a) numerical approach (Eq. 1 in Fig. 1), b) analytical mono-exponential two-parameter model (Eq. 2, Fig. 1), and c) three-parameter offset model (Eq. 3, Fig. 1). The analytical models were fit applying a Levenberg-Marquardt algorithm of nonlinear estimation. The measured data were expressed with 95% confidence intervals. R^2^ was used to describe the quality of the resulting fit. Significance of the results was tested by applying two-tailed paired Student’s t-test.

All data were acquired on 1.5T clinical whole-body scanner. The Eurospin Test Object TO5 Contrast phantom was used for initial validation of the T2p-SSFP technique and comparison of accuracy of investigated models. Phantom data were acquired with 9 different T2 preparation times (TE from 0 to 150ms). Acquisition parameters: matrix 240x240, slice thickness 5mm, FOV 230^2^mm^2^, TR/TE 4.4/2.2ms, flip angle 60°.

**Figure 2:**
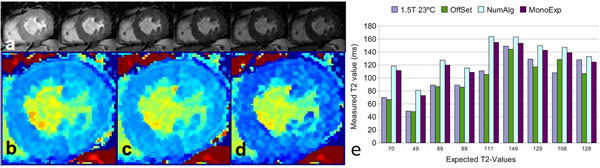
a) Raw images acquired at *TE* = 0ms, 20ms, 35ms, 50ms, 65ms, 80ms correspondingly; b) T2 map acquired with numerical approach, c) T2 map acquired with mono-exponential model, d) T2 map acquired with offset model; e) comparison of three models on phantom data

The identified optimal parameter set was applied for in vivo measurements. T2-prepared experiments with 6 preparation times (TE = 0,20,35,50,65,80ms) were performed consecutively within a single breathhold (Fig. 2a). Data was acquired during end diastole applying single-slice SSFP pulse sequence with 11 startup echoes. To avoid motion artifacts, acquisition of each T2 preparation delay was split over two subsequent cardiac cycles and parallel imaging acceleration of 2 was used. Image parameters: number of phase encodings per cardiac cycle 29, partial Fourier acquisition with centric reordering, flip angle 60°, TR/TE 3.5/1.75ms, FOV 380^2^mm^2^, matrix 288x288, slice thickness 10mm, bandwidth 515.7Hz/pixel, acquisition window 102ms and scan duration of 12RR cycles. Image registration was performed to compensate for residual respiratory motion.

## Results

The phantom data (Fig. 2e) revealed that the offset model performs best for fitting the data. A non significant (p = 0.35) deviation from the recorded T2 values was observed, whereas mono-exponential (p = 0.0027) and numerical (p = 0.00038) models significantly overestimate the data. In vivo measurements showed a significant (p = 0.003) reduction in the calculated T2 value for the offset model. T2-mapping applying the offset model yields a mean T2 value of 33.7ms (Fig. 2d) vs 41.6ms (Fig. 2c) and 40.9ms (Fig. 2b) for the mono-exponential and numerical models.

## Conclusions

The phantom data showed superior performance of the offset model for T2 quantification as compared to the mono-exponential and numerical model for T2p-SSFP. The respective quantification of myocardial T2 values in vivo yielded a lower value as previously reported. This may indicate that applying simple mono-exponential decay model may overestimate resulting T2 value in myocardium.

## Funding

EU FP7 REBORNE

Project Reference 241879.

